# Exercise elicits mitonuclear protein imbalance and UPR^mt^ in the liver of mice with obesity

**DOI:** 10.1007/s13105-026-01229-4

**Published:** 2026-09-01

**Authors:** Matheus Biscaro Rocha, Izabela Monteiro de Araujo, Saulo Gabriel do Nascimento Della Coleta, Scylas J. A. Junior, Sidney F. Peres, Renata R. Braga, Rafael S. Brícola, Fernanda S. Carneiro, Barbara M. Crisol, Raul G. da Costa, Marcelo A. S. Mori, Dennys E. Cintra, Adelino S. R. da Silva, José R. Pauli, Eduardo Rochete Ropelle

**Affiliations:** 1 Laboratory of Molecular Biology of Exercise (LaBMEx), School of Applied Sciences, University of Campinas (UNICAMP), Limeira, São Paulo, 13484-350 Brazil; 2 Sorbonne Université, Inserm, Centre de Recherche en Myologie, Paris, UMRS974 France; 3 Department of Biochemistry and Tissue Biology, University of Campinas, Campinas, Brazil; 4 Laboratory of Nutritional Genomics, University of Campinas(UNICAMP), Limeira, São Paulo Brazil; 5 Postgraduate Program in Rehabilitation and Functional Performance, Ribeirão Preto Medical School, University of São Paulo (USP), Ribeirão Preto, São Paulo, Brazil; 6 School of Physical Education and Sport of Ribeirão Preto, University of São Paulo, Ribeirão Preto, São Paulo, Brazil; 7 National Institute of Science and Technology for Obesity and Diabetes, Campinas, Brazil; 8 CEPECE - Center of Research in Sport Sciences, School of Applied Sciences, University of Campinas (UNICAMP), Limeira, São Paulo, 13484-350 Brazil; 9 Laboratory of Molecular Biology of Exercise, University of Campinas, Limeira, 13484 - 350 SP Brazil

**Keywords:** Exercise; Mitonuclear protein imbalance; UPRmt; MASLD; Obesity; Liver; Mitochondrial proteostasis

## Abstract

Mitochondrial dysfunction plays a critical role in the development of metabolic dysfunction-associated steatotic liver disease (MASLD). It has been proposed that mitochondrial unfolded-protein response (UPR^mt^) activation improves mitochondrial function in the liver. Growing evidence demonstrates that physical exercise effectively prevents and treats MASLD. However, the effects of exercise on UPR^mt^ activation in the liver are unknown. Thus, we investigated the impact of aerobic training on the mechanisms involved in mitochondrial quality control in the liver in a mouse model of obesity. Liver transcript data from a genetic reference panel of BXD isogenic mice revealed a negative correlation between UPR^mt^-related genes and hepatic triacylglycerol content. In addition, the liver UPR^mt^ markers were strongly associated with several mitochondrial-related genes in the hepatic tissue of BXD mice and humans. Notably, 4 weeks of aerobic exercise strongly impacted the liver metabolism, preventing intrahepatic lipid accumulation in HFD-fed mice. Physical exercise boosted the NAD-biosynthesis pathway, elicited the mitonuclear protein imbalance, stimulated the protein content of UPR^mt^-markers, including CLpP, Lonp1, and Yme1L1, and improved the mitochondrial proteostasis and function in the liver in HFD-fed mice. Thus, our findings link the mitonuclear protein imbalance and UPR^mt^ activation in the liver to mitochondrial proteostasis and MASLD prevention in response to physical exercise.

**Supplementary Information:**

The online version contains supplementary material available at https://doi.org/10.1007/s13105-026-01229-4.

## Introduction

Hepatic steatosis is a common condition caused by the abnormal accumulation of fatty acids in the liver, which can progress to nonalcoholic steatohepatitis, fibrosis, and, ultimately, cirrhosis [1]. Beyond the genetic factors, obesity and lifestyle, including overnutrition and sedentarism, are the main factors related to steatosis. Hepatic steatosis is generally associated with other asymptomatic clinical conditions, such as low-grade inflammation, insulin resistance, dyslipidemia, and aberrant hepatic lipogenesis [2]. These characteristics make hepatic steatosis a public health challenge for the coming decades.

Mitochondrial dysfunction in hepatocytes promotes incomplete fatty acid oxidation, contributing to hepatic fatty acid accumulation, making mitochondrial dysfunction a critical target for steatosis treatment [2]. Disruption of mitochondrial proteostasis in hepatic tissue is closely associated with mitochondrial dysfunction, fatty liver accumulation, and metabolic dysfunction-associated steatotic liver disease (MASLD) [3, 4]. Mitochondrial dysfunction is a complex phenomenon involving multiple factors, including the accumulation of unfolded or misfolded proteins in the mitochondria, which generates a proteotoxic environment within the organelle [5]. In this scenario, the conserved mechanism of eukaryotic cells (e.g., mitonuclear protein imbalance) is considered an important cellular phenomenon for maintaining mitochondrial proteostasis [6]. The imbalance between nuclear (nDNA)- and mitochondrial (mtDNA)-encoded OXPHOS proteins triggers a retrograde signal from mitochondria to nDNA that upregulates mitochondrial chaperones (HSP60, HSP72, TRAP1, and PHB) and proteases (CLPP, Yme1L1, Lonp1, Oma1, and PARL), which are capable of restoring the mitochondrial proteostasis and function [7]. The upregulation of these specific mitochondrial chaperones and proteases is called mitochondrial Unfolded Protein Response (UPR^mt^) [8, 9].

The mitonuclear protein imbalance and UPR^mt^ are activated by the intracellular NAD^+^ abundance in a SIRT1-dependent mechanism [3]. Interestingly, oral treatment with the NAD^+^ precursor nicotinamide riboside (NR) induced mitonuclear protein imbalance and activated the UPR^mt^, leading to improved hepatic mitochondrial function and reversal of hepatic steatosis in a preclinical model of MASLD [3]. Similar data were found by using other NAD^+^ boosters, including the PARP inhibitors [4, 10]. These data illustrate the critical role of NAD^+^ metabolism in hepatic function, highlighting mitonuclear protein imbalance and UPR^mt^ activation as potential mechanisms for preventing or treating MASLD.

Physical exercise is an effective non-pharmacological strategy for reversing hepatic steatosis. Although the exact mechanism by which exercise reduces liver fat content remains unclear, a growing body of evidence suggests that hepatocyte mitochondrial function plays a pivotal role in this process [11]. In recent years, we have demonstrated that exercise improves mitochondrial function in skeletal muscle and in the hypothalamic tissue by inducing mitonuclear protein imbalance and UPR^mt^ activation in different mouse models [12–15]. Thus, we hypothesized that exercise might elicit the mitonuclear protein imbalance and UPR^mt^ in the liver, improving mitochondrial proteostasis and contributing to the prevention of hepatic steatosis.

## Materials and methods

### Bioinformatics analysis

Human and mouse databases were accessed on GeneNetwork (http://www.genenetwork.org). The human database was GTEXv5 Human Liver RefSeq (Sep15) RPKM log2 (Human liver_GTEx v5), as previously described [16]. Mouse transcriptomic analyses were performed using liver data from an independent cohort of BXD recombinant inbred mouse strains fed a high-fat diet (BXD HFD Liver Affy Mouse Gene) [17]. Importantly, these BXD mice were distinct from the Swiss mice used in the exercise experiments. The BXD database was widely used to investigate the genetic regulation of metabolic phenotypes and disease susceptibility. To estimate hepatic UPR^mt^ activity, a UPR^mt^ score was calculated based on the mean expression of four UPR^mt^-related genes (*Hspd1*,* Yme1L1*,* Lonp1*, and *Clpp*) across 42 HFD-fed BXD strains. Data for blood glucose levels during the intraperitoneal glucose tolerance test (area under the curve) of 18-week-old males were obtained from Andreux and colleagues [17], and data for hepatic triacylglycerol were obtained from Jha and colleagues [18]. GraphPad Prism (version 9.5.1) was used for Pearson’s correlation analyses, and heat map graphs were generated using Gene-E online software (https://software.broadinstitute.org/GENE-E/).

### Animals

8 weeks old Swiss male mice were obtained from the University of Campinas (UNICAMP) animal facility. The mice were distributed into three groups: control, HFD (high-fat diet), and HFD-TR (trained high-fat diet). An a priori sample size calculation was performed based on the expected effect size and variance of the primary outcome, indicating that 10 animals per group would provide adequate statistical power. Each mouse was housed in a cage under controlled temperature conditions (21 °C ± 1.0 °C). The animals were maintained on a 12:12-hour light-dark cycle. The HFD and HFD-TR groups were fed a high-fat diet (HFD) for 12 weeks, as previously described [19]. However, after the first 8 weeks of HFD consumption, the HFD-TR group began the exercise protocol. The experiments were approved by the Ethics Committee of the University of Campinas under protocol number 5694-1/2021. The number of animals analyzed varied according to the experimental procedure (*n* = 5–10 per group), and the exact sample size for each analysis is indicated in the corresponding figure legends. The animals had free access to water and to either conventional chow or a high-fat diet. Food consumption was measured weekly, and intake values were converted to kcal.

### Exercise protocol

The mice in the exercised group were acclimated to treadmill running for 5 consecutive days (10 min per day at 8 m/min) to reduce stress. After the acclimatization period, the animals underwent the incremental load test [20] to determine the maximal speed of exhaustion (MSE). The test was initiated at 6 m/min and increased by 3 m/min every 3 min until exhaustion. The exhaustion point for each animal was defined as the point at which the mouse failed to respond to gentle encouragement to run and remained at the back of the treadmill for more than 15 s. The maximum speed achieved, running distance, and time to exhaustion were monitored. This protocol provided us with individual aerobic capacity and the intensity of effort during training. Next, the mice underwent 4 weeks of running exercise (60 min per session) at an intensity corresponding to 60% of their MSE, as previously described [20–22]. The exercise protocol was performed between 12:00 and 14:00. The incremental test was also performed after the exercise protocol.

### Tissue sampling

Forty-eight hours after the last exercise session and after a 4-hour fasting period, the animals were euthanized by anesthetic deepening, using an intraperitoneal injection of ketamine hydrochloride (10%) (300 mg/Kg) and xylazine hydrochloride (2%) (30 mg/Kg), followed by cervical dislocation. Different adipose tissue depots were collected and weighed. The samples from hepatic tissue were also collected for Western blotting, histological, and RT-qPCR analyses. For the Western blotting analysis, the liver samples were homogenized in RIPA buffer. The samples were centrifuged at 12.000 RPM for 40 min. The supernatant portion was used for the protein concentration determination through the bicinchoninic acid method. After adding the Laemmli buffer, the samples were stored at -80 °C.

### Cholesterol and glucose analysis

Total cholesterol levels in the serum were measured using commercial kits (Laborlab, São Paulo, SP, Brazil). Serum samples were collected during liver extraction following decapitation, 48 h after physical exercise, and after a 4-hour fasting period.

The fasting glucose measurement was performed after a 12-hour fasting period. Blood samples were obtained by tail snip, using surgical scissors. A single blood drop was used for the glucose measurement (Accu-Check Active, Roche, Switzerland).

### Histological analyses (HE)

Liver histological analyses were performed based on previously established procedures [23]. Briefly, liver samples were collected following euthanasia, rapidly frozen in pre-cooled isopentane, and stored at − 80 °C until further processing. Cryosections (10 μm thickness) were obtained using a cryostat (Leica CM1850, Germany), placed on glass slides, and stained with hematoxylin and eosin (H&E) according to standard histological procedures. Images were acquired using Motic Images Plus 3.0 software for histological assessment. Representative images were obtained from each experimental group and analyzed to evaluate hepatic morphology and the presence of lipid accumulation.

### Western blotting

The Western blotting was performed as previously described [24]. Antibodies anti-YME1L1 (11510-AP) from Proteintech; anti-LONP1(bs-4245R) from BIOSS (Boston, MA); anti-SIRT1 (2028 S), anti-α-Tubulin and (2144s) anti-Vinculin (4650s) from Cell Signaling; anti-NMNAT1 (ab45652), anti-CLPP (ab124822), anti-OXPHOS (MS604-300) from ABCAM; and anti-PEPCK (sc-32879), anti-HSP60 (sc-13115), anti-NMNAT3 (sc-390433), anti-PBEF (NAMPT) (sc-67020) and anti-GAPDH (sc-47724) from Santa Cruz Biotechnology (Santa Cruz, CA, USA) were used. All antibodies used were diluted 1:1000. Ponceau staining was employed to control the total loading of protein in each gel. The bands were quantified by Image J software (1.54 g version), and the values were normalized by the respective loading control group.

### Mitonuclear protein imbalance determination

Mitonuclear protein imbalance was determined based on the stoichiometric relationship between mitochondrial DNA (mtDNA)-encoded and nuclear DNA (nDNA)-encoded OXPHOS proteins, as previously described [25]. To assess mitonuclear protein imbalance in the liver, the protein levels of MTCO1, an mtDNA-encoded OXPHOS subunit, were compared with nuclear-encoded OXPHOS proteins obtained from the same blot. The resulting ratio was used as an indirect indicator of mitonuclear protein imbalance.

### UPR^mt^ scores

UPR^mt^ activation is commonly assessed by measuring the mRNA or protein levels of mitochondrial chaperones and proteases. To provide an integrated estimate of the overall UPR^mt^ response, we calculated a composite UPR^mt^ score based on the arithmetic mean of the expression levels of selected UPR^mt^ markers, herein referred to as the UPRmt score. For this study, we used the arithmetic mean of the gene expression of *Hspd1*, *Yme1L1*, *Lonp1*, and *Clpp* from the liver of BXD mice to obtain the UPR^mt^ score. For the Western blot experiment, the UPR^mt^ score was obtained through arithmetic means from the relativized values of the proteins CLPP, HSP60, Lonp1, and YME1L1 from the liver of Swiss mice.

### RT-qPCR

The real-time PCR was performed as previously described [26]. Real-time PCRs were performed using 300 ng cDNA and 0.3 µM of the following primers:

*Scd1* forward: GAGGCCTGTACGGGATCATA and reverse: CCGAAGAGGCAGGTGTAGAG;

*Fasn* forward: AGCTTCGGCTGCTGTTGGAAGT and reverse: TCGGATGCCTCTGAACCACTCACA;

*Acta2* forward: CCCAGACATCAGGGAGTAATGG and reverse: TCTATCGGATACTTCAGCGTCA;

*Fn1* forward: ACTGGATGGGGTGGGAAT and reverse: GGAGTGGCACTGTCAACCTC;

*Col1a1* forward: CCTCAGGGTATTGCTGGACAAC and reverse:

CAGAAGGACCTTGTTTGCCAGG;

*Atf4* forward: AACCTCATGGGTTCTCCAGCGA and reverse:

CTCCAACATCCAATCTGTCCCG;

*Ccn2* forward: TGCGAAGCTGACCTGGAGGAAA and reverse:

CCGCAGAACTTAGCCCTGTATG;

*Tgfb1* forward: TGATACGCCTGAGTGGCTGTCT and reverse:

CACAAGAGCAGTGAGCGCTGAA;

*Chop* forward: GGAGGTCCTGTCCTCAGATGAA and reverse: GGAGGTCCTGTCCTCAGATGAA

The primers were synthesized by Exxtend (Campinas, SP, Brazil) and SYBR® Green PCR Master Mix (Applied Biosystems, Carlsbad, CA, USA). The cycling parameters were: 10 min at 95 °C followed by 40 × 15s cycles at 95 °C and 60 s at 60 °C. The relative mRNA content was determined after normalization with Ywhaz using the ΔΔCt method. Each set of primers was designed to recognize unique regions of gene sequences.

### High-resolution respirometry analysis

The high-resolution respirometry analysis was performed using Oxygraph-2k (Oroboros Instruments, Austria). For the analysis, 20 mg of frozen samples were homogenized in MAS buffer (220 mM mannitol, 70 mM sucrose, 10 mM KH₂PO₄, 5 mM MgCl_2_, 2 mM HEPES, and 1 mM EGTA), and added to each chamber, with the measurement performed at 37 °C. The complex I respiratory capacity was measured using NADH (100 µM), the combined complexes I + II was measured with the addition of succinate (10 mM), and the isolation of complex II respiration was measured with the addition of rotenone (0.5 µM). To assess the β-oxidation capacity, octanoylcarnitine (OCT) (0.2 mM) was used, and to measure the maximum respiratory capacity, a titration of carbonylcyanide p-trifluoromethoxy phenyl-hydrazone (FCCP) (0.5 μm) was added. At last, the non-mitochondrial respiration was measured by the addition of antimycin A (2.5 μm).

### NAD⁺ quantification

Hepatic NAD⁺ levels were determined as previously described by Kanamori et al. (2018). Briefly, approximately 20 mg of liver tissue was homogenized in 10% trichloroacetic acid, followed by sonication and centrifugation. The resulting pellet was used for protein quantification to normalize NAD⁺ levels, whereas the supernatant was used for NAD⁺ determination. Samples were analyzed in a 96-well plate using a fluorescence microplate reader for 60 min, with excitation and emission wavelengths of 544 and 590 nm, respectively. NAD⁺ levels were normalized to protein content and expressed as nmol/mg ptn.

### Statistical analysis

GraphPad Prism (version 9.5.1) was used for statistical analysis. All results were expressed as a mean ± standard deviation (SD). The data were analyzed by " t-Student” when comparing two groups and analysis of variance (ANOVA), one-way analysis when comparing more than two groups, followed by the Tukey post-test. Pearson’s correlation analysis was also employed. The statistical significance adopted was *p* < 0.05.

## Results

### UPR^mt^ in the hepatic tissues of mice and humans

**Fig. 1:**
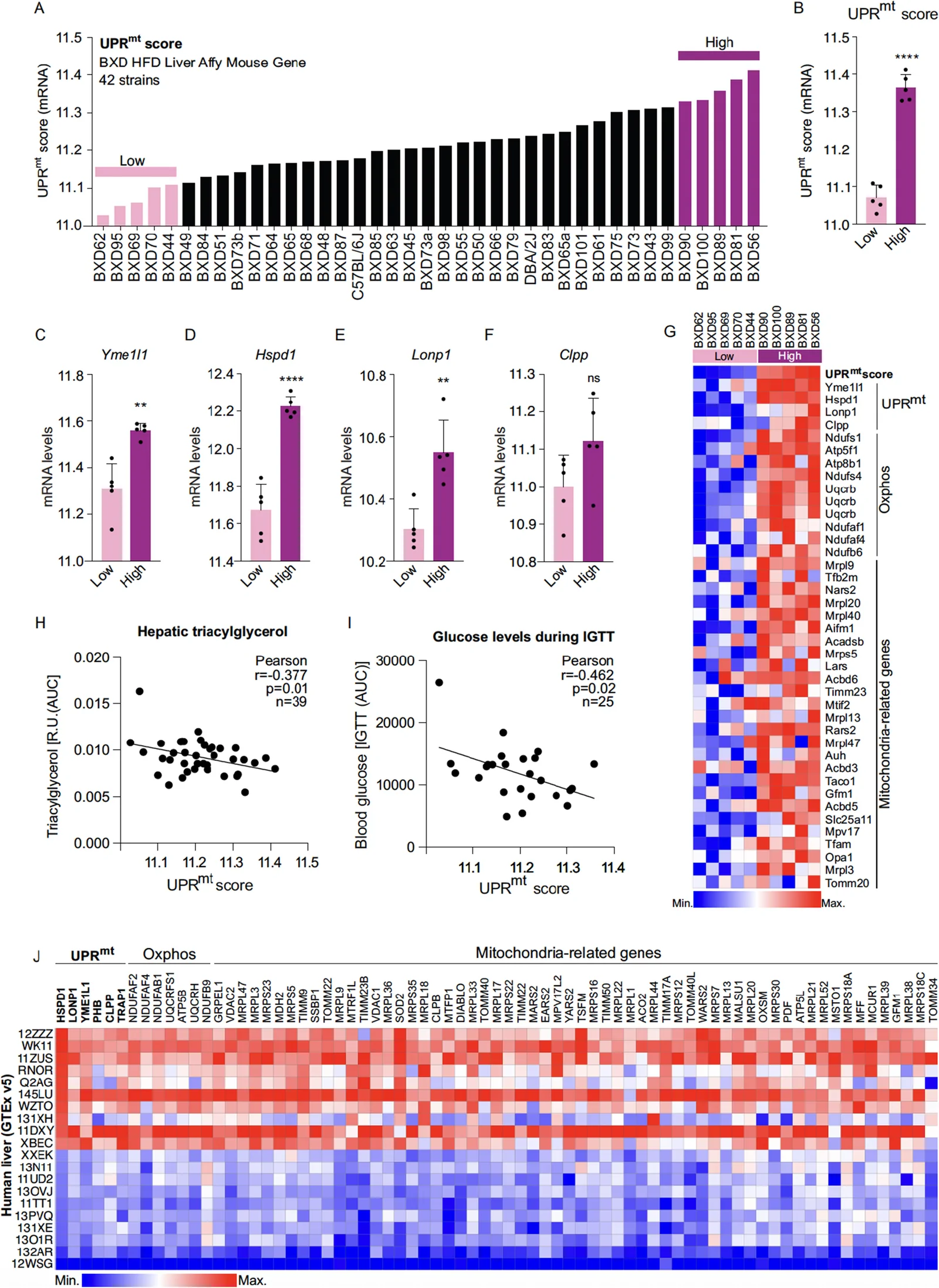
Association between UPR^mt^ and mitochondrial markers in mice and humans. (**A**) UPR^mt^ score of the liver from 42 BXD mouse strains fed HFD. (**B**) 5 strains with low and 5 with high UPR^mt^ scores in the liver. (**C**-**F**) UPR^mt^ markers in low and high strains. (**G**) Heatmap showing UPR^mt^, Oxphos, and other mitochondria-related genes in the hepatic tissue of BXD mice (Low x High) (*n* = 10). Pearson’s correlation between UPR^mt^ and (**H**) Hepatic triacylglycerol and (**I**) Glucose levels during the intraperitoneal glucose tolerance test in BXD mice fed HFD. **, *p* < 0.01 vs. Low and ****, *p* < 0.0001 vs. Low. **(J**) Heatmap showing UPR^mt^, Oxphos, and other mitochondria-related genes in the liver of humans. (*n* = 20)

Firstly, to explore the association between hepatic UPR^mt^ and metabolic dysfunction, we performed transcriptome analysis using liver data from an independent cohort of BXD mice fed on a high-fat diet (BXD HFD Liver Affy Mouse Gene) [17]. The BXD database is a murine reference panel composed of several recombinant inbred isogenic mouse strains widely used to investigate the genetic control of metabolism and diseases [17]. Using 42 strains fed on a high-fat diet, we ranked these mouse families by the hepatic UPR^mt^ score, composed of the mean of the gene expression of 4 UPR^mt^ components (*Hspd1*,* Yme1L1*,* Lonp1*, and *Clpp*) (Figs. 1A and B). Based on this ranking, five BXD mouse strains with low and high UPR^mt^ scores in the liver were selected (Figs. 1A and B). The BXD strains with very high UPR^mt^ scores in the liver displayed high levels of UPR^mt^ markers, as expected (Figs. 1C-F), but also presented high levels of OXPHOS- and several other mitochondria-related genes in the hepatic tissue (Fig. 1G), and an opposite pattern was also observed in BXD strains with very low UPR^mt^ scores in the liver (Fig. 1G). Using the whole cohort of BXD mice, we found a moderate but significant negative correlation between UPR^mt^ score and hepatic triacylglycerol content (Fig. 1H) and between UPR^mt^ score and area under the curve during the glucose tolerance test (Fig. 1I).

Next, liver transcripts from a heterogeneous human population (*n* = 134) were also analyzed [16]. These individuals were ranked based on the gene encoding the human HSP60 protein, *HSPD1* (data not shown). Next, we selected the ten lowest and ten highest individuals expressing the *HSPD1* gene in the liver (Fig. 1J). In addition to *HSPD1*, these groups exhibited high levels of other UPR^mt^ markers, including *LONP1*,* YME1L1*,* PHB*,* CLPP*, and *TRAP1* (Fig. 1J). Interestingly, individuals with high levels of UPR^mt^-related genes also presented high levels of OXPHOS- and several other mitochondria-related genes in the hepatic tissue (Fig. 1J). An opposite signature was observed in subjects with the lowest UPR^mt^ markers in the liver (Fig. 1J), showing a clear association between UPR^mt^-related genes and mitochondria-related genes in humans. This initial exploration confirmed a positive association between UPR^mt^ markers and mitochondrial metabolism in the liver of obese mice and, for the first time, the positive association between UPR^mt^ markers and mitochondria-related genes in a heterogeneous population of humans.

### Exercise improves liver metabolism in obese mice

**Fig. 2:**
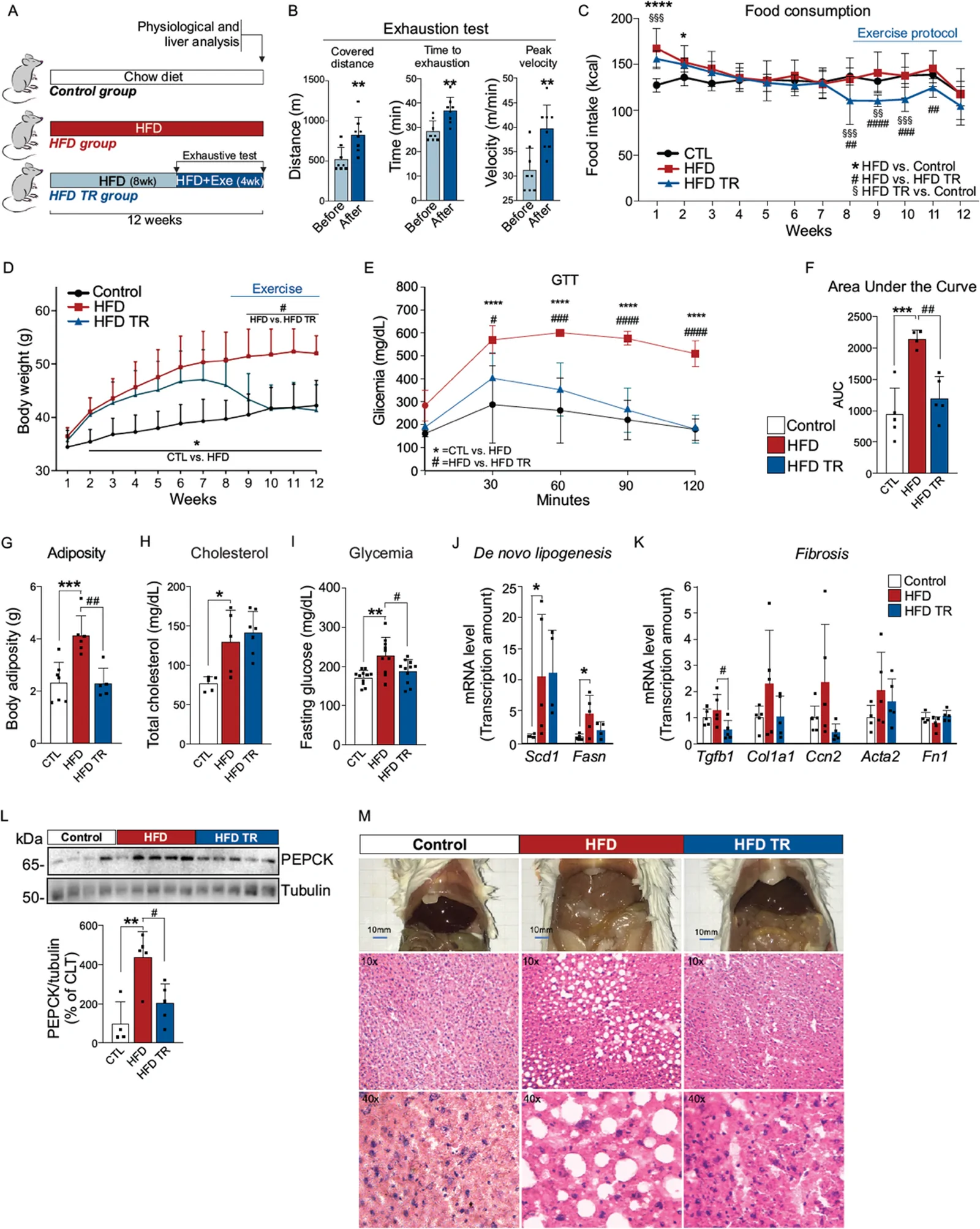
Effects of exercise on the physiological parameters and the liver of mice fed HFD. (**A**) Experimental design. (**B**) Trained group performance comparison before and after the exercise protocol. (*n* = 8). **, *p* < 0.01. (**C**) Food consumption of the animals during 12 weeks of protocol. (*n* = 6–10). #, *p* < 0.05 HFD vs. HFD TR. (**D**) Body weight of the animals during 12 weeks of protocol. (*n* = 6–10). *, *p* < 0.05 CTL vs. HFD and #, *p* < 0,05 HFD vs. HFD TR. (**E**) Glucose tolerance test (GTT). (*n* = 5–7). ****, *p* < 0.0001 CTL vs. HFD and #, ###, ####, *p* < 0.05, *p* < 0.001 and *p* < 0.0001 HFD vs. HFD TR. (**F**) Area under the curve (AUC) of the GTT. (*n* = 5–7). *, *p* < 0.001 CTL vs. HFD and ##, *p* < 0.01 HFD vs. HFD TR. (**G**) Body adiposity was determined by the sum of epididymal, mesenteric, retroperitoneal, and subcutaneous white adipose tissue weights. (*n* = 5–7). ***, *p* < 0.001, ##, *p* < 0.01. (**H**) Total serum cholesterol from mice fed HFD. (*n* = 5–7). *, *p* < 0.05. (I) Fasting glycemia. (*n* = 10–11). **, *p* < 0.01 and #, *p* < 0.05. (**J**-**K**) *De novo* lipogenesis- and fibrosis-related genes in the liver. (*n* = 4–5). *, *p* < 0.05. (L) PEPCK protein content in the liver of mice. (*n* = 4–5). **p < 0.01 and #, *p* < 0.05. (M) Macroscopic and microscopic images from the liver of mice

Next, we determined the effects of exercise training on liver metabolism and UPRmt markers. For this, Swiss mice were fed an HFD for 8 weeks and then subjected to 4 weeks of treadmill aerobic exercise (Fig. 2A). This exercise protocol promoted a positive physiological adaptation, as demonstrated by the exhaustive running test (Fig. 2B). Lower caloric intake was observed in obese mice during the physical exercise protocol (Fig. 2C). While HFD treatment increased body weight, adiposity, total serum cholesterol, fasting glycemia, and impaired glucose tolerance (Fig. 2D–I), aerobic exercise reduced body weight, adiposity, fasting glycemia, and improved glucose tolerance, as demonstrated by both the GTT and the area under the curve (AUC) analyses (Fig. 2D–G and I). However, exercise did not significantly alter total serum cholesterol levels (Fig. 2H). Gene expression analysis of liver samples demonstrated that HFD treatment increased Scd1 and Fasn expression, whereas aerobic exercise did not significantly alter these markers of de novo lipogenesis (Fig. 2J). Regarding fibrosis-related genes, Tgfb1 expression was significantly increased in HFD-fed mice and reduced following aerobic exercise. No statistically significant differences were observed in Col1a1, Ccn2, Acta2, or Fn1 expression (Fig. 2K). The HFD consumption increased phosphoenolpyruvate carboxykinase (PEPCK) protein content, a critical enzyme involved in hepatic gluconeogenesis, while exercise training reduced it (Fig. 2L), corroborating the fasting glucose data. Under visual inspection, HFD treatment increased the fat accumulation in the liver, while exercise training promoted a strong effect, reducing the liver fat content (Fig. 2M, upper panels). This data was confirmed using histological analysis (Fig. 2M, lower panels) and demonstrated that the adopted exercise protocol promoted consistent benefits for hepatic metabolism beyond fitness improvement.

#### Obesity alters mitochondrial proteostasis in the liver

**Fig. 3:**
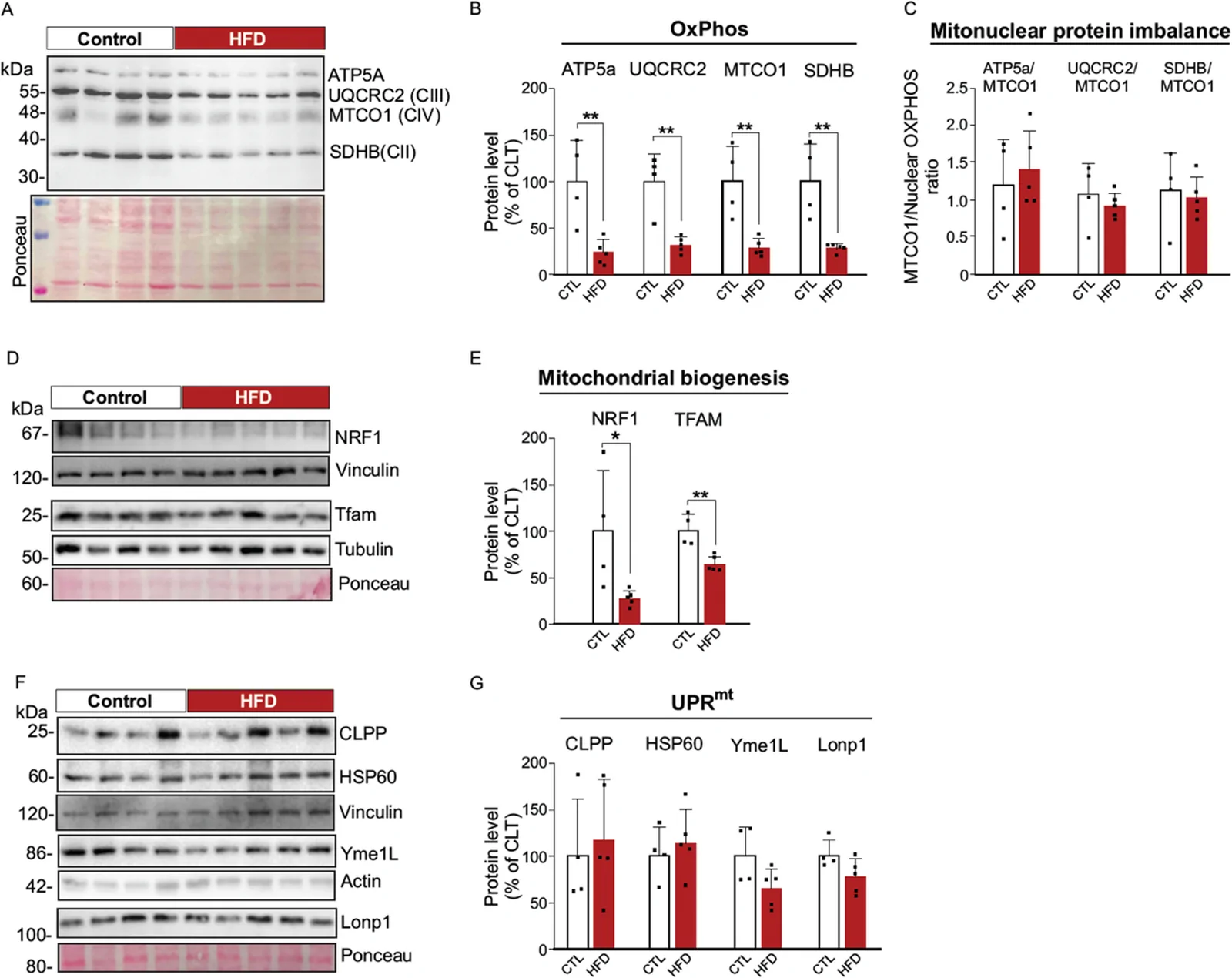
Effects of HFD treatment on mitochondrial proteostasis in the hepatic tissue. (**A** and **B**) Western blot and quantification of OXPHOS components in the liver. (**C**) Mitonuclear protein imbalance was evaluated as the ratio between nuclear-encoded OXPHOS proteins and MTCO1 blot shown in Fig. 3A. (**D **and **E**) Protein content of NRF1 and TFAM in the liver. (**F** and **G**) Protein content of UPR^mt^ markers in the liver. *, *p* < 0.05 and **, *p* < 0.01. (*n* = 4–5)

We next sought to determine the effects of HFD on mitochondrial proteostasis in the hepatic tissues. The Western blot analysis demonstrated that HFD reduced the protein content of the OXPHOS components, including Ubiquinol-Cytochrome C Reductase Core Protein 2 (UQCRC2 - CIII), Cytochrome c oxidase subunit 1 (MTCO1 - CIV), and Succinate Dehydrogenase Complex Subunit B (SDHB - CII), and the component of ATPase, ATP synthase subunit alpha (ATP5A) (Fig. 3A and B). No difference was found in terms of the mitonuclear protein imbalance between control and HFD-treated mice (Fig. 3C).

The HFD treatment reduced the protein content of critical mitochondrial biogenesis markers, such as the Nuclear Respiratory Factor 1 (NRF1) and TFAM (transcription factor A, mitochondrial) (Fig. 3D and E). However, no statistical difference was observed in the protein content of UPR^mt^ markers, caseinolytic mitochondrial matrix peptidase proteolytic subunit (CLPP), Heat Shock Protein 60 (HSP60), ATP-dependent zinc metalloprotease (YME1L1), and Lon protease 1, mitochondrial (Lonp1) (Fig. 3F and G). These data demonstrate that the HFD alters the mitochondrial proteostasis, without affecting the mitonuclear balance or the UPR^mt^ markers.

#### Exercise elicits the mitonuclear protein imbalance and stimulates UPR^mt^ in the liver of mice with obesity

**Fig. 4:**
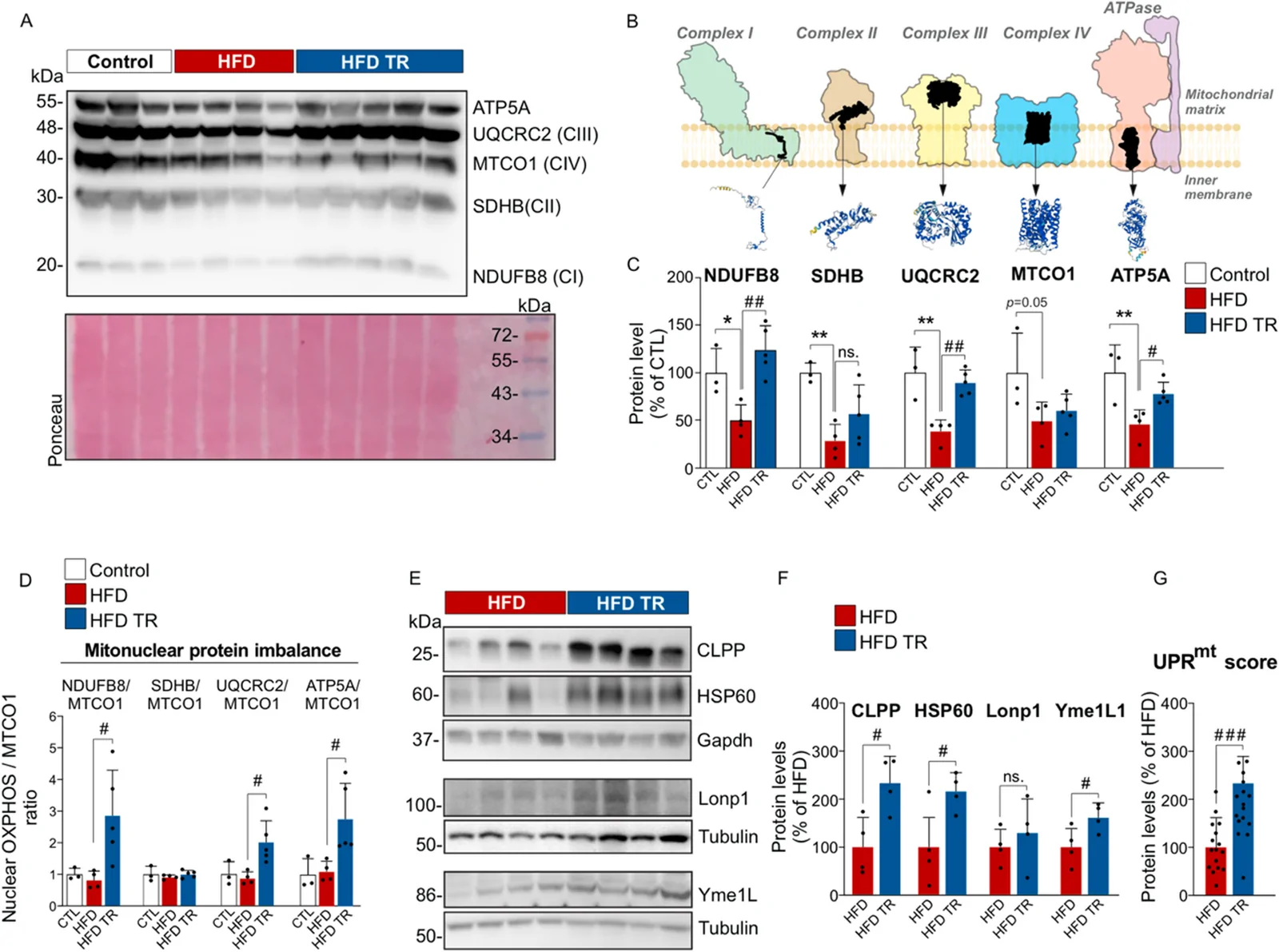
Effects of exercise on mitochondrial proteostasis and UPR^mt^ markers. (**A **and **C**) Western blot and quantification of the protein content of OXPHOS components in the liver. (*n* = 3–5). (**B**) Illustration of each OXPHOS component on its respective complexes. (**D**) Mitonuclear protein imbalance was evaluated as the ratio between nuclear-encoded OXPHOS proteins and MTCO1 blot shown in Fig. 4A. (**E** and **F**) Western blot and quantification of the protein content of UPR^mt^ markers in the hepatic tissue (*n* = 4). (**G**) UPR^mt^ score, #, *p* < 0,05 ##, *p* < 0.01, ###, *p* < 0.001, and ####, *p* < 0.0001

Thereafter, we evaluated the effects of chronic physical exercise on mitochondrial proteostasis in the livers of mice with obesity. Western blot analysis revealed that physical exercise increased the protein content of components of complex I (NDUFB8), complex III (UQCRC2), and ATPase (ATP5A) in the liver (Fig. 4A-C). Interestingly, physical exercise promoted the stoichiometric imbalance between nDNA- and mtDNA-encoded OXPHOS subunits, confirming the presence of the mitonuclear protein imbalance in the liver of exercised mice (Fig. 4D).

These findings were accompanied by the accumulation of proteins related to UPR^mt^ in the hepatic tissue, including the mitoproteases CLPP and Yme1L1 and the chaperone HSP60 (Fig. 4E and F). However, no differences were found in the gene expression of *Chop* and *Atf4*, transcription factors associated with stress-responsive pathways, including the mammalian UPR^mt^. (Figure S1). Collectively, these data indicate that aerobic exercise training is associated with increased UPR^mt^ markers and improved mitochondrial proteostasis in the livers of obese mice.

#### Exercise restores the NAD biosynthesis pathway in the liver of mice with obesity

**Fig. 5:**
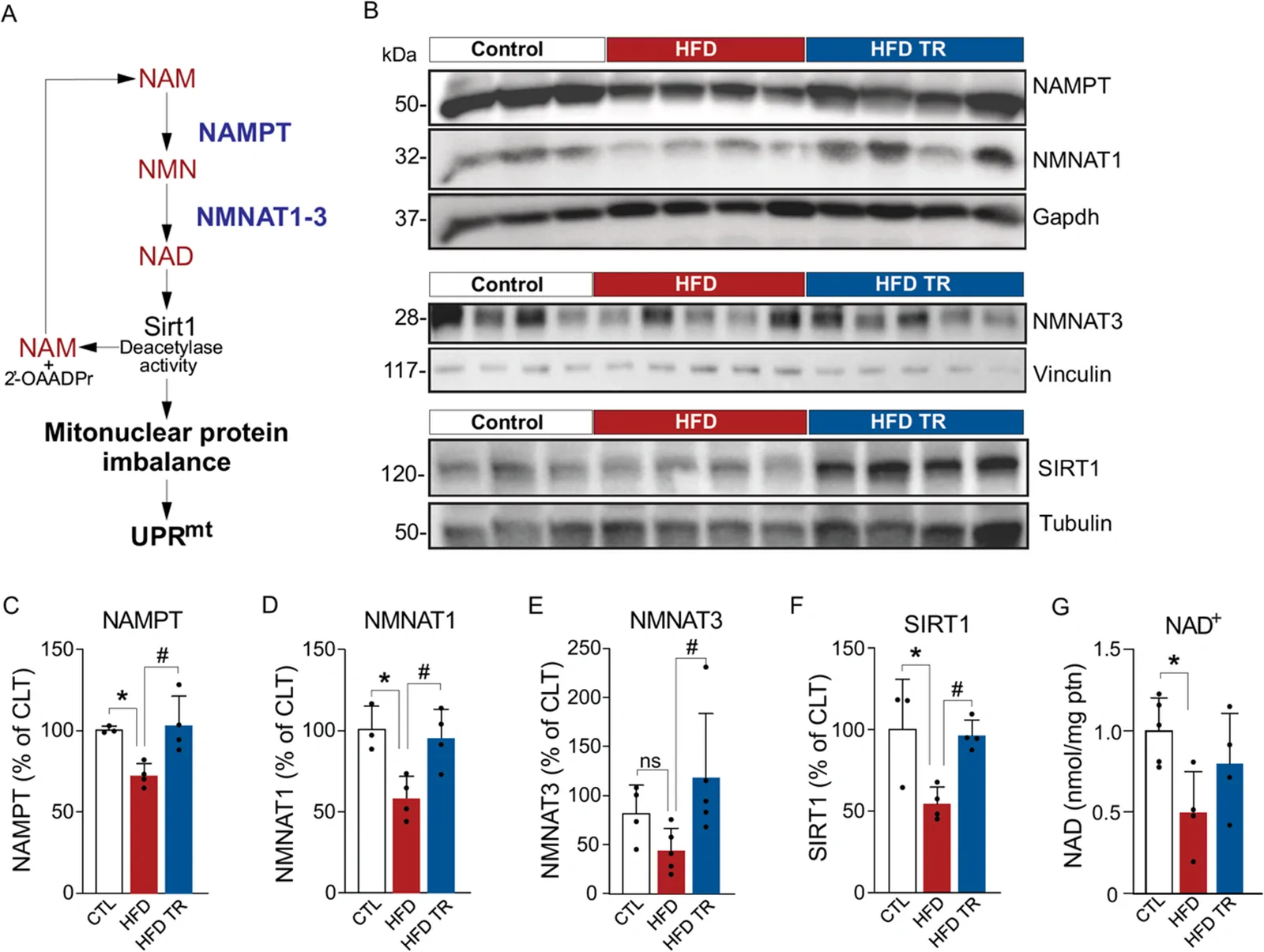
Effects of exercise on the NAD biosynthesis pathway and on mitonuclear protein imbalance in the liver of mice. (**A**) Schematic view of the NAD biosynthesis pathway. (**B**) Protein content of NAMPT, NMNAT1, NMNAT3 (*n* = 3–5) and SIRT1 (*n* = 3–4). (**C**-**F**) Protein quantification. (**G**) NAD^+^ measurement in the hepatic tissue (*n* = 5 − 4). *, *p* < 0.05, and #, *p* < 0.05

Once the mitonuclear protein imbalance and UPR^mt^ are, at least in part, dependent on the NAD^+^ levels and the SIRT1 activation [3, 4] (Fig. 5A), we next sought to determine the NAD biosynthesis pathway and SIRT1 protein content in our model. Initially, we found a significant reduction in the protein content of the key enzymes involved in the control of NAD^+^ synthesis (NAMPT and NMNAT1) in the liver of mice with obesity, when compared to the control group (Fig. 5B-E). However, the exercise training increased NAMPT, NMNAT1, and NMNAT3 protein contents in the hepatic tissue, compared to the HFD group (Fig. 5B-E). We also monitored the protein content of SIRT1. While HFD treatment suppressed hepatic SIRT1 protein levels, exercise restored the SIRT1 content in the liver (Fig. 5B and F).

Measurement of hepatic NAD⁺ levels confirmed that HFD treatment significantly reduced NAD⁺ content compared with the control group. Exercise training was associated with a 37% increase in hepatic NAD⁺ levels relative to the HFD group; however, this difference did not reach statistical significance. Accordingly, hepatic NAD⁺ levels in exercised HFD mice were no longer significantly different from those of control animals. Taken together, these findings demonstrate that HFD disrupts the NAD⁺ biosynthesis pathway, as evidenced by reduced abundance of key biosynthetic enzymes, lower SIRT1 protein levels, and decreased hepatic NAD⁺ content. Chronic exercise training restored the abundance of proteins involved in the NAD⁺ biosynthesis pathway (NAMPT, NMNAT1 and NMNAT3) and SIRT1, while showing a trend toward recovery of hepatic NAD⁺ levels.

#### Exercise improves mitochondrial respiratory capacity in the livers of obese mice

**Fig. 6:**
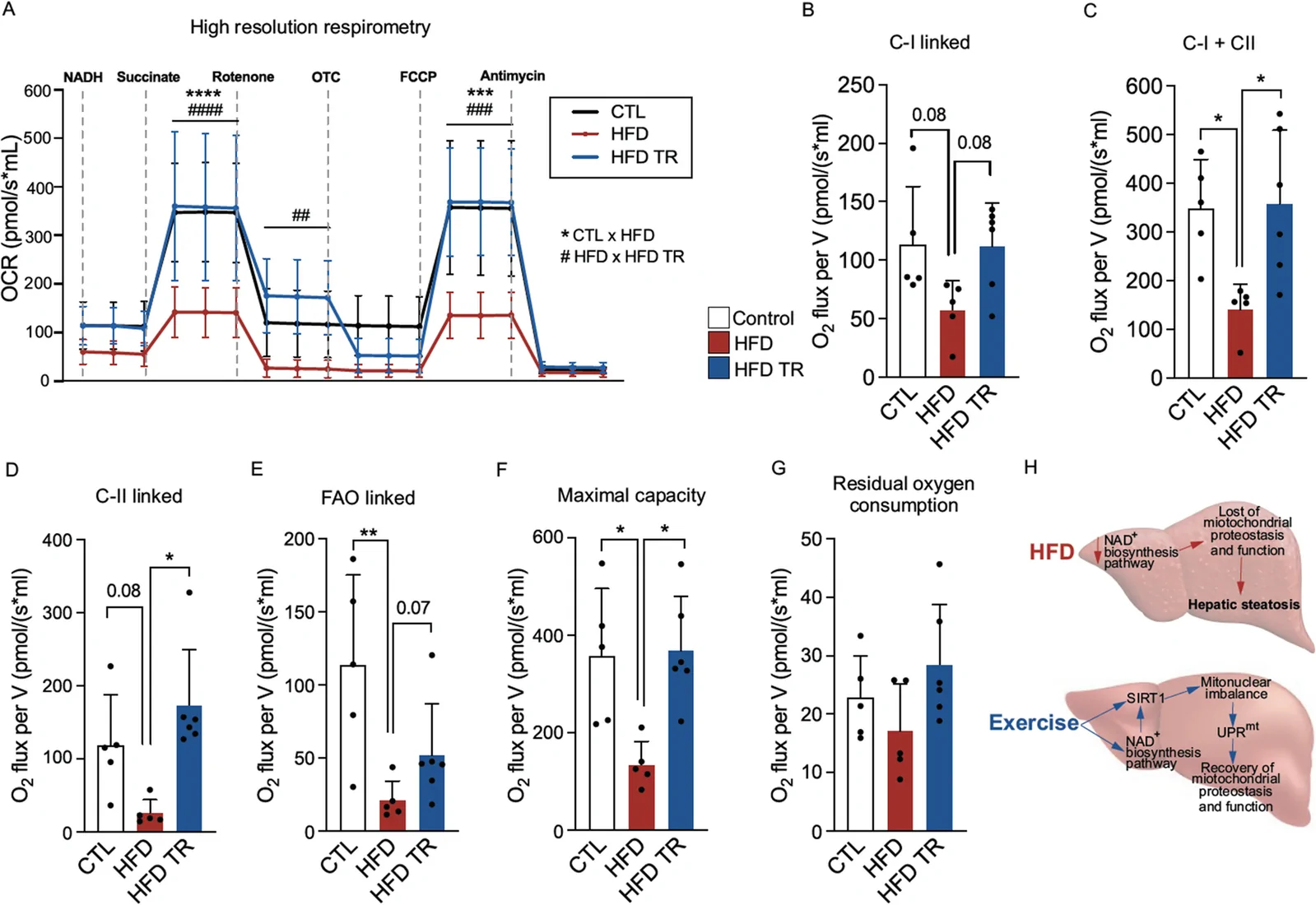
Effects of exercise on mitochondrial respiration in the liver of mice. (**A**) High-resolution respirometry in the liver of mice. (*n* = 5–6) *, difference between CTL vs. HFD, and #, difference between HFD vs. HFD + TR, *p* < 0.05. (**B**) Oxygen consumption linked to Complex (1) (**C**) Oxygen consumption linked to the Complex 1 plus Complex (2) (**D**) Oxygen consumption linked to Complex 2. (**E**) Oxygen consumption linked to fatty acid oxidation. (**F**) Maximal capacity of oxygen consumption. (**G**) Oxygen consumption linked to the residual respiration. (*n* = 5–6) *, *p* < 0.05, and **, *p* < 0.01. (**H**) Illustration summarizing the main findings regarding the effects of HFD and physical activity in the liver of mice with obesity

To assess whether the observed mitonuclear protein imbalance and UPRmt result in functional changes in mitochondrial respiratory capacity, we performed high-resolution respirometry. Accordingly, a significant reduction in mitochondrial respiration was observed in the liver of obese mice, with a higher respiration observed in the trained group (Fig. 6A). Although a trend toward reduced complex I-linked respiration was observed in the obese group compared to both control and trained (Fig. 6B), the stimulation of both complexes I and II, as well as the isolated Complex II respiration, showed a lower respiratory capacity in the obese group, as well as an increase of C-II respiration in the trained group (Fig. 6C and D).

Notably, the obese mice exhibited a lower fatty acid oxidation (FAO)-linked respiration compared to the control group, while trained mice showed a tendency to restore the fatty acid oxidation capacity in the hepatic tissue (*p* = 0.07) (Fig. 6E). Importantly, the maximum respiratory capacity was significantly reduced in the hepatic tissue of obese mice; however, the maximum respiratory capacity returned to the control levels in the liver of trained mice (Fig. 6F), without differences in the residual oxygen consumption between groups (Fig. 6G). Together, these findings demonstrate that exercise is associated with improved mitochondrial respiratory capacity in the liver of obese mice, as evidenced by lower C-II-linked, FAO-linked, and maximal respiration, whereas exercise can restore mitochondrial respiration and enhance oxidative capacity.

## Discussion

Hepatic steatosis, a condition that can progress to various other diseases [1], has become increasingly prevalent alongside rising obesity rates. Therefore, identifying effective treatment strategies is crucial. A meta-analysis study demonstrated that physical exercise is an effective intervention against MASLD in humans [27]. Consistent with these findings, the present study provides robust evidence that the preventive effects of aerobic training on MASLD are associated with mitonuclear protein imbalance and UPR^mt^ activation in mouse hepatic tissue.

Intracellular NAD^+^ levels are reduced in fatty liver [4], which can be recovered with NAD^+^ boosting strategies such as NR supplementation and PARP inhibitors [4, 10]. These studies support the hypothesis that increased NAD+ availability promotes UPRmt activation and facilitates the recovery of hepatic tissue from MASLD. Interestingly, physical exercise is a physiological NAD^+^ booster in both humans [28] and mice [14]. To confirm this information, we analyzed the NAD^+^ synthesis pathway in the hepatic tissue. First, we found that HFD treatment suppressed the protein levels of two critical enzymes involved in NAD^+^ synthesis (NAMPT and NMNAT1) and reduced the NAD levels in hepatic tissue. Next, our data revealed that aerobic training increased the protein levels of NAMPT, NMNAT1, and NMNAT3 in the livers of HFD-treated mice. Although the trained animals showed an increase of approximately 37% in the hepatic NAD+ levels compared to the obese group, statistical significance was not observed.

SIRT1 is the most studied protein in the sirtuin family and is known as a metabolic modulator across various tissues [29]. The lack of SIRT1 in mice can induce MASLD [30], and moderate overexpression of SIRT1 can recover the liver from these conditions [31]. Another study showed a decrease in SIRT1 content in the livers of rats fed an HFD, which was restored by calorie restriction [32]. Moreover, SIRT1 expression was upregulated in the liver of diabetic mice [33] and zebrafish [34] models of MASLD following exercise. Our experiments showed reduced SIRT1 content in the HFD group, which could lead to additional impairments in hepatic tissue metabolism. We also observed a significant accumulation of SIRT1 in the livers of trained animals, supporting the hypothesis that exercise contributes to SIRT1 regulation in the liver.

AMPK is another key metabolic sensor activated by exercise and has been implicated in the regulation of mitochondrial quality control. Previous studies have demonstrated that AMPK increases intracellular NAD+ availability, thereby promoting SIRT1 activation and mitochondrial adaptive responses [35, 36], including UPRmt activation. Although AMPK signaling was not directly evaluated in the present study, it may have contributed to the exercise-induced improvements in hepatic mitochondrial proteostasis observed in our model.

A NAD^+^- boosting strategy could activate mitonuclear protein imbalance and UPR^mt^ via SIRT1 activation, thereby conferring several benefits on the livers of obese mice [3]. Interestingly, other studies have shown the presence of mitonuclear protein imbalance and UPR^mt^ activation induced by exercise in skeletal muscle [12–14]. However, this mechanism remained unknown in hepatic tissue. Thus, our results indicate the presence of mitonuclear protein imbalance and UPR^mt^ markers, including CLPP, LONP1, and YME1L1, in response to physical activity. The activation of mitonuclear protein imbalance and the UPR^mt^ may be associated with increased expression of proteins involved in the NAD^+^ biosynthesis pathway, increased SIRT1 content, and potentially the activation of other exercise-responsive signaling pathways, such as AMPK, in the livers of trained mice [36–41]. However, how physical activity influences the levels of these proteins was not determined in the present study and warrants further investigation.

Although studies on the metabolic effects of UPR^mt^ activation in mammals are increasing, the field still yields divergent findings. In genetic models, mice lacking CLPP are protected from diet-induced obesity and insulin resistance [42]. Mice heterozygous for Hsp60 displayed mitochondrial dysfunction and insulin resistance in the hypothalamus [43]. In mouse models of obesity, the high-fat and high sucrose diet (HFHS) consumption reduced the mitochondrial function in the liver of C57BL6j mice without affecting the mRNA levels of UPR^mt^-related genes [3]. However, HFD treatment for 20 weeks increased *Lonp1* and *Hsp60* gene expression in the liver of mice. Conversely, long-term HFD treatment (6 months) suppressed HSP60 protein levels in the hepatic tissue of mice [44]. Curiously, metformin treatment induced Lonp1 and HSP60 protein levels and improved mitochondrial parameters in a mouse model of diabetes [45]. In the current study, 12 weeks of HFD treatment did not alter the protein levels of 4 UPR^mt^ components: CLPP, HSP60, Yme1L1, and Lonp1, and the gene expression of *Chop* and *Atf4*. However, 4 weeks of physical exercise increased the protein content of CLPP, HSP60, and Yme1L1, without affecting *Chop* and *Atf4* gene expression in the liver. The increase in the content of these proteases (CLPP and Yme1L1) and the chaperone (HSP60) was accompanied by the recovery of mitochondrial proteostasis, improved mitochondrial respiratory function, including fatty acid oxidation-linked respiration, and overall metabolic improvement of the liver in our model.

Importantly, our high-resolution respirometry analyses further demonstrated that aerobic exercise partially restored fatty acid oxidation-linked mitochondrial respiration in HFD-fed mice. Improved hepatic fatty acid oxidative capacity has been associated with enhanced mitochondrial function and metabolic homeostasis in MASLD [36]. Although we did not evaluate the expression of additional genes involved in fatty acid oxidation, these functional data indicate an improvement in hepatic mitochondrial fatty acid oxidative capacity, which may have contributed to the recovery of mitochondrial function and metabolic homeostasis observed in the exercised animals.

In addition to improving mitochondrial proteostasis, aerobic exercise significantly reduced hepatic *Tgfb1* expression, suggesting attenuation of profibrotic signaling. Although *Atf4*, *Chop*, *Ccn2*, and *Col1a1* expression did not differ significantly between groups, they exhibited similar expression profiles, with higher values in HFD-fed mice and lower values after exercise training. These findings are consistent with the overall improvement in hepatic metabolism and mitochondrial function observed in the exercised animals.

Limitations of the study: Although our study provides robust evidence that physical exercise increases the protein levels of several UPR^mt^ markers in liver tissue, some limitations should be mentioned. Exercise-induced weight loss may represent an important contributing factor to the improvement in hepatic metabolism, potentially influencing several of the outcomes evaluated in this study. Further studies are needed to clarify whether physical exercise alone can modulate the NAD+ biosynthesis pathway or stimulate UPRmt markers in hepatic tissue independently of changes in body weight, and whether these effects are also observed in the livers of healthy animals. In the NAD^+^ measurement assay, we observed that obesity affected the levels of this coenzyme in the liver; although training demonstrated a clear trend toward increased hepatic NAD^+^ levels, statistical significance was not reached. The rise in values within the trained group, combined with the small sample size, likely prevented the observation of a statistically significant difference. Another important consideration is that, although increased SIRT1 protein levels were observed in exercised animals, this does not necessarily indicate enhanced enzymatic activity of this molecule.

Collectively, our findings show that aerobic exercise prevents hepatic lipid accumulation and is associated with improved mitochondrial proteostasis and respiratory function in HFD-fed mice. These adaptations were accompanied by increased expression of proteins involved in the NAD^+^ biosynthesis pathway, higher SIRT1 protein content, mitonuclear protein imbalance, and increased UPR^mt^ markers, supporting the hypothesis that activation of these mitochondrial quality control pathways contributes to the beneficial effects of exercise on hepatic metabolism (Fig. 6H). Finally, our study provides new insights into how physical exercise ameliorates mitochondrial dysfunction in obesity-induced hepatic diseases.

## Supplementary information

Below is the link to the electronic supplementary material.


Supplementary Material 1


## Data Availability

The datasets generated and/or analyzed during the current study are partially publicly available. Human data were obtained from the Genotype-Tissue Expression project (GTEx v5), and mouse data were accessed via GeneNetwork. Additional datasets were obtained from previously published studies. Experimental data generated in this study are available from the corresponding author upon reasonable request.

## References

[1] Cohen JC, Horton JD, Hobbs HH (2011) Human fatty liver disease: Old questions and new insights. Sci (1979) 332(6037):1519–1523 10.1126/science.1204265 PMC3229276 21700865

[2] Sunny NE, Parks EJ, Browning JD, Burgess SC (2011) Excessive hepatic mitochondrial TCA cycle and gluconeogenesis in humans with nonalcoholic fatty liver disease. Cell Metab 14(6):804–810 22152305 10.1016/j.cmet.2011.11.004 PMC3658280

[3] Gariani K, Menzies KJKJ, Ryu D et al (2016) Eliciting the mitochondrial unfolded protein response by nicotinamide adenine dinucleotide repletion reverses fatty liver disease in mice. Hepatology 63(4):1190–1204 26404765 10.1002/hep.28245 PMC4805450

[4] Gariani K, Ryu D, Menzies KJ et al (2017) Inhibiting poly ADP-ribosylation increases fatty acid oxidation and protects against fatty liver disease. J Hepatol 66(1):132–141 27663419 10.1016/j.jhep.2016.08.024

[5] Mouchiroud L, Houtkooper RH, Moullan N et al (2013) The NAD+/sirtuin pathway modulates longevity through activation of mitochondrial UPR and FOXO signaling. Cell 154(2):430 23870130 10.1016/j.cell.2013.06.016 PMC3753670

[6] Qureshi MA, Haynes CM, Pellegrino MW (2017) The mitochondrial unfolded protein response: Signaling from the powerhouse. J Biol Chem 292(33):13500–13506 28687630 10.1074/jbc.R117.791061 PMC5566509

[7] Yi HS, Chang JY, Shong M (2018) The mitochondrial unfolded protein response and mitohormesis: A perspective on metabolic diseases. J Mol Endocrinol 61(3):R91–R105 30307158 10.1530/JME-18-0005 PMC6145237

[8] Yoneda T, Benedetti C, Urano F, Clark SG, Harding HP, Ron D (2004) Compartment-specific perturbation of protein handling activates genes encoding mitochondrial chaperones. J Cell Sci 117(18):4055–4066 15280428 10.1242/jcs.01275

[9] Zhao Q, Wang J, Levichkin IV, Stasinopoulos S, Ryan MT, Hoogenraad NJ (2002) A mitochondrial specific stress response in mammalian cells. EMBO J 21(17):4411–4419 12198143 10.1093/emboj/cdf445 PMC126185

[10] Mukhopadhyay P, Horváth B, Rajesh M et al (2017) PARP inhibition protects against alcoholic and non-alcoholic steatohepatitis. J Hepatol 66(3):589–600 27984176 10.1016/j.jhep.2016.10.023

[11] Morris EM, Meers GME, Koch LG et al (2016) Aerobic capacity and hepatic mitochondrial lipid oxidation alters susceptibility for chronic high-fat diet-induced hepatic steatosis. Am J Physiol Endocrinol Metab 311(4):E749–E760 27600823 10.1152/ajpendo.00178.2016 PMC5241560

[12] Cordeiro AV, Rafael Brícola BS, Renata Braga BR et al (2020) Aerobic Exercise Training Induces the Mitonuclear Imbalance and UPRmt in the Skeletal Muscle of Aged Mice. Journals Gerontology: Ser A. 10.1093/gerona/glaa059/5805478 32173728

[13] Cordeiro AV, Peruca GF, Braga RR et al (2020) High-intensity exercise training induces mitonuclear imbalance and activates the mitochondrial unfolded protein response in the skeletal muscle of aged mice. Geroscience. 10.1007/s11357-020-00246-5 32737758 PMC8190321

[14] Crisol BM, Veiga CB, Braga RR et al (2019) NAD+ precursor increases aerobic performance in mice. Eur J Nutr. 10.1007/s00394-019-02089-z 31494696

[15] Gaspar RS, Katashima CK, Crisol BM et al (2023) Physical exercise elicits UPRmt in the skeletal muscle: The role of c-Jun N-terminal kinase. Mol Metab 78. 10.1016/J.MOLMET.2023.101816 PMC10590869 37821006

[16] Lonsdale J, Thomas J, Salvatore M et al (2013) The Genotype-Tissue Expression (GTEx) project. Nat Genet 45(6):580–585 23715323 10.1038/ng.2653 PMC4010069

[17] Andreux PA, Williams EG, Koutnikova H et al (2012) Systems genetics of metabolism: The use of the BXD murine reference panel for multiscalar integration of traits. Cell 150(6):1287–1299 22939713 10.1016/j.cell.2012.08.012 PMC3604687

[18] Jha P, McDevitt MT, Gupta R et al (2018) Systems Analyses Reveal Physiological Roles and Genetic Regulators of Liver Lipid Species. Cell Syst 6(6):722–733e6 29909277 10.1016/j.cels.2018.05.016 PMC6054463

[19] Gaspar RC, Veiga CB, Bessi MP et al (2019) Unsaturated fatty acids from flaxseed oil and exercise modulate GPR120 but not GPR40 in the liver of obese mice: a new anti-inflammatory approach. J Nutr Biochem 66:52–62 30771734 10.1016/j.jnutbio.2018.12.003

[20] Ferreira JCB, Rolim NPL, Bartholomeu JB, Gobatto CA, Kokubun E, Brum PC (2007) Maximal lactate steady state in running mice: Effect of exercise training. Clin Exp Pharmacol Physiol 34(8):760–765 17600553 10.1111/j.1440-1681.2007.04635.x

[21] Massett MP, Matejka C, Kim H (2021) Systematic review and meta-analysis of endurance exercise training protocols for mice. Front Physiol 12:782695. 10.3389/fphys.2021.782695 PMC8691460 34950054

[22] PICOLI CDC et al (2018) Peak Velocity as an Alternative Method for Training Prescription in Mice. v. 9, n. February, pp. 1–5 10.3389/fphys.2018.00042 PMC5808179 29467664

[23] Pinto AP, Muñoz VR, Tavares MEA et al (2023) Combined physical exercise reverses the reduced expression of Bmal1 in the liver of aged mice. Life Sci 312. 10.1016/J.LFS.2022.121175 36414092

[24] Silva VRR, Micheletti TO, Pimentel GD et al (2014) Hypothalamic S1P/S1PR1 axis controls energy homeostasis. Nat Commun 5:4859 25255053 10.1038/ncomms5859

[25] Houtkooper RH, Mouchiroud L, Ryu D et al (2013) Mitonuclear protein imbalance as a conserved longevity mechanism. Nature 497(7450):451–457 23698443 10.1038/nature12188 PMC3663447

[26] Katashima CK, de Oliveira Micheletti T, Braga RR et al (2022) Evidence for a neuromuscular circuit involving hypothalamic interleukin-6 in the control of skeletal muscle metabolism. Sci Adv 8(30). 10.1126/SCIADV.ABM7355 PMC9337767 35905178

[27] Babu AF, Csader S, Lok J et al (2021) Positive effects of exercise intervention without weight loss and dietary changes in nafld-related clinical parameters: A systematic review and meta-analysis. Nutrients 13(9). 10.3390/nu13093135 PMC8466505 34579012

[28] de Guia RM, Agerholm M, Nielsen TS et al (2019) Aerobic and resistance exercise training reverses age-dependent decline in NAD+ salvage capacity in human skeletal muscle. Physiol Rep 7(12). 10.14814/phy2.14139 PMC6577427 31207144

[29] Mariani S, Fiore D, Basciani S et al (2015) Plasma levels of SIRT1 associate with non-alcoholic fatty liver disease in obese patients. Endocrine 49(3):711–716 25358448 10.1007/s12020-014-0465-x

[30] Xu F, Gao Z, Zhang J et al (2010) Lack of SIRT1 (mammalian sirtuin 1) activity leads to liver steatosis in the SIRT1+/- mice: A role of lipid mobilization and inflammation. Endocrinology 151(6):2504–2514 20339025 10.1210/en.2009-1013 PMC2875813

[31] Pfluger PT, Herranz D, Velasco-Miguel S, Serrano M, Tschöp MH (2008) Sirt1 protects against high-fat diet-induced metabolic damage. Proc Natl Acad Sci U S A 105(28):9793–9798 18599449 10.1073/pnas.0802917105 PMC2474520

[32] Deng XQ, Chen LL, Li NX (2007) The expression of SIRT1 in nonalcoholic fatty liver disease induced by high-fat diet in rats. Liver Int 27(5):708–715 17498258 10.1111/j.1478-3231.2007.01497.x

[33] Liu HW, Kao HH, Wu CH (2019) Exercise training upregulates SIRT1 to attenuate inflammation and metabolic dysfunction in kidney and liver of diabetic db/db mice. Nutr Metab (Lond) 16(1):1–10 30988688 10.1186/s12986-019-0349-4 PMC6446356

[34] Zou Y, Chen Z, Sun C et al (2021) Exercise intervention mitigates pathological liver changes in NAFLD zebrafish by activating SIRT1/AMPK/NRF2 signaling. Int J Mol Sci 22(20):1–17 10.3390/ijms222010940 PMC8536011 34681600

[35] Steinberg GR, Hardie DG (2023) New insights into activation and function of the AMPK. Nat Rev Mol Cell Biol 24(4):255–272. 10.1038/s41580-022-00547-x 36316383

[36] STEINBERG GR et al (2025) Integrative metabolism in MASLD and MASH: Pathophysiology and emerging mechanisms. J Hepatol v 83(2):584–595 10.1016/j.jhep.2025.02.033 40032040

[37] Camacho RC, Donahue EP, James FD, Berglund ED, Wasserman DH (2006) Energy state of the liver during short-term and exhaustive exercise in C57BL/6J mice. Am J Physiol Endocrinol Metab 290(3):E405–E408. 10.1152/ajpendo.00385.2005 16219665

[38] HUGHEY CC et al (2023) Exercise training adaptations in liver glycogen and glycerolipids require hepatic AMP-activated protein kinase in mice. n September:2026 10.1152/ajpendo.00289.2023 PMC11193517 37938177

[39] Wu B, Zhang Z, Xu C, Zhao J (2025) Aerobic exercise improved liver steatosis by modulating miR-34a-mediated PPARα/SIRT1-AMPK signaling pathway. PloS one 20(11):e0333872. 10.1371/journal.pone.0333872 PMC12611108 41223206

[40] Wu M, Zhao A, Yan X, Gao H, Zhang C, Liu X, Luo Q, Xie F, Liu S, Shi D (2022) Hepatic AMPK signaling dynamic activation in response to REDOX balance are sentinel biomarkers of exercise and antioxidant intervention to improve blood glucose control. eLife 11:e79939. 10.7554/eLife.79939 PMC9645808 36155132

[41] Yu Y, Zhu G, Zhang Z, Wang H, Zeng L, Xia J, Liu X, Fang C, Liu S, Yang Y, Yu L (2025) Exercise ameliorates nonalcoholic fatty liver disease by reducing the IGFBP5 to IGF1 ratio to activate AMPK pathway. Scientific reports 15(1):23083. 10.1038/s41598-025-07857-y PMC12219279 40593070

[42] Becker C, Kukat A, Szczepanowska K et al (2018) CLPP deficiency protects against metabolic syndrome but hinders adaptive thermogenesis. EMBO Rep 19(5). 10.15252/EMBR.201745126 PMC5934779 29588285

[43] Kleinridders A, Lauritzen HPMM, Ussar S et al (2013) Leptin regulation of Hsp60 impacts hypothalamic insulin signaling. J Clin Invest 123(11):4667–4680 24084737 10.1172/JCI67615 PMC3809782

[44] Weng SW, Wu JC, Shen FC et al (2023) Chaperonin counteracts diet-induced non-alcoholic fatty liver disease by aiding sirtuin 3 in the control of fatty acid oxidation. Diabetologia 66(5):913–930 36692509 10.1007/s00125-023-05869-9

[45] Docrat TF, Nagiah S, Naicker N, Baijnath S, Singh S, Chuturgoon AA (2020) The protective effect of metformin on mitochondrial dysfunction and endoplasmic reticulum stress in diabetic mice brain. Eur J Pharmacol 875. 10.1016/J.EJPHAR.2020.173059 32131023

