# Supplementary material for: Exercise elicits mitonuclear protein imbalance and UPRmt in the liver of mice with obesity
Source: J Physiol Biochem. 2026 Sep 1;82(1):88. doi: 10.1007/s13105-026-01229-4 (PMC13534197; doi:10.1007/s13105-026-01229-4)
Supplement: Supplementary file 1 [file 13105_2026_1229_MOESM1_ESM.pdf]

SupplementaryfigureS1

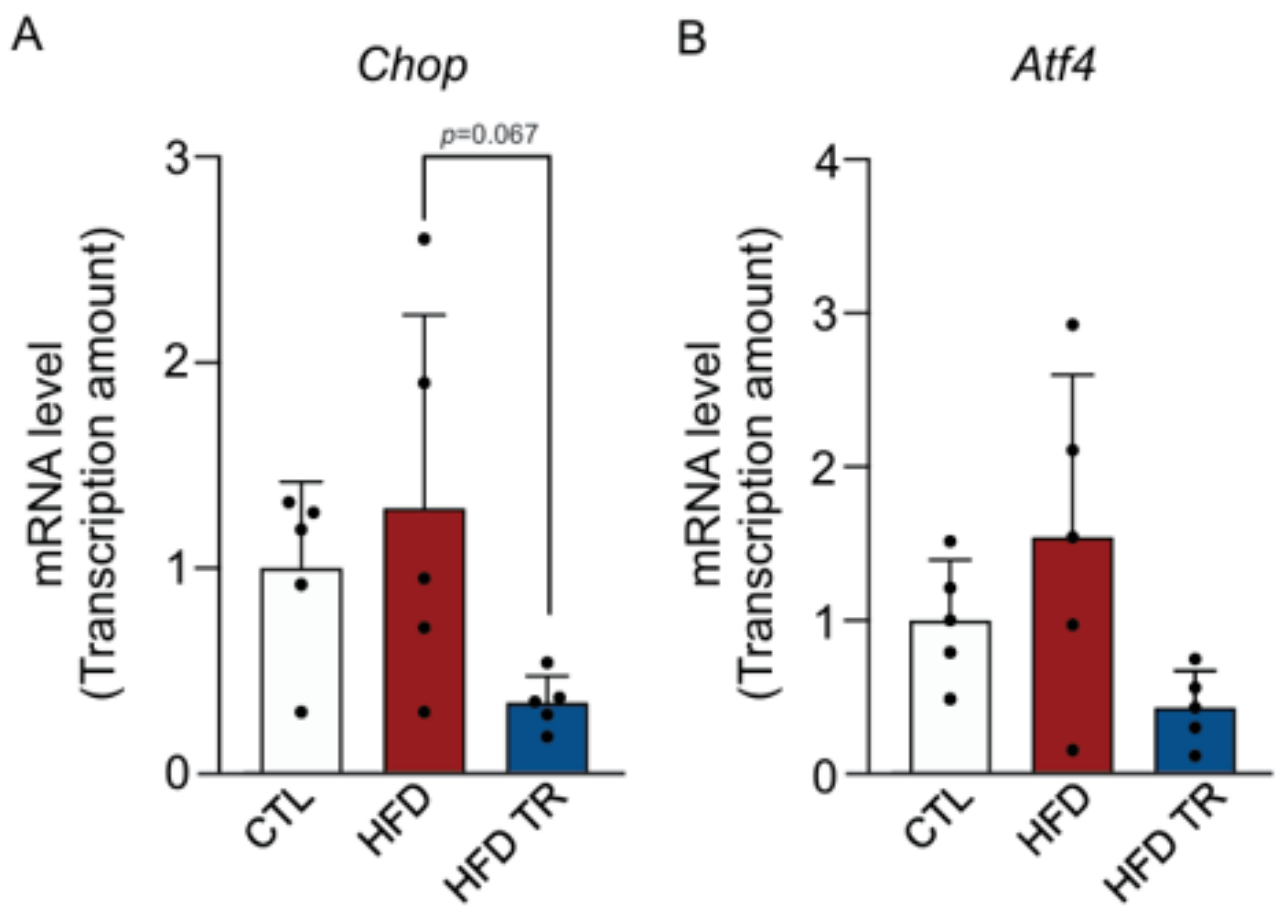

Figure S1. Effects of a high-fat diet and exercise on hepatic expression of stress-responsive transcription factor genes. (A–B) Relative mRNA expression of stress-responsive transcription factor genes, determined by RT-qPCR in the liver (n = 4–5)
